# Percutaneous Liver Biopsy after Living Donor Liver Transplantation Resulting in Fulminant Hepatic Failure: The First Reported Case of Hepatic Compartment Syndrome

**DOI:** 10.1155/2010/273578

**Published:** 2010-04-08

**Authors:** Nicholas N. Nissen, Stephen A. Geller, Andrew Klein, Steve Colquhoun, David Yamini, Tram T. Tran, Benjamin Weinberg, Julie Winn, Fred Poordad

**Affiliations:** ^1^Hepatology and Liver Transplantation, Cedars-Sinai Medical Center, 8635 W. 3rd Street #1060-W, Los Angeles, CA 90048, USA; ^2^23961 Calle De La Magdalena, Laguna Hills, CA 92653, USA; ^3^2601 East Main St., Suite 102, Ventura, CA 93003, USA

## Abstract

A 28-year-old female who underwent live donor liver transplantation 3 years prior presented after percutaneous liver biopsy with abdominal and shoulder pain, nausea, vomiting, and elevated liver enzymes. Computed tomography (CT) showed an intrahepatic and subcapsular hematoma. There was a progressive increase in liver enzymes, bilirubin, and INR and a decline in hemoglobin. Subsequent CT imaging revealed flattening of the portal vein consistent with compression by the enlarging hematoma. Liver failure ensued and the patient required urgent retransplantation. The explant demonstrated ischemic necrosis of greater than 90% of the liver parenchyma. We report this case of “Hepatic Compartment Syndrome” leading to fulminant hepatic failure.

## 1. Introduction

Percutaneous liver biopsy is routinely performed in the posttransplant setting for elevated liver enzymes of unclear etiology. Complications requiring hospitalization occur in 1.4%–3.2% of percutaneous biopsies [1, 2]. Although asymptomatic bleeding can be detected by ultrasound in up to 23% of cases, significant bleeding occurs in only 0.078%–0.32% of percutaneous liver biopsies [3–5]. Mortality is rare, seen in 1 in 10,000–12,000 cases [5, 6]. 

We report a case of intrahepatic and subcapsular bleeding after liver biopsy causing compression of the portal vein and massive ischemic hepatic necrosis. We postulate that the expanding hematoma led to elevated intrahepatic pressures that in turn resulted in diminished hepatic perfusion and ischemia. This “Hepatic Compartment Syndrome” led to fulminant hepatic failure requiring emergent liver transplantation. This etiology of fulminant hepatic failure has not been previously reported.

## 2. Case Report

A 28-year-old female underwent adult-to-adult live donor liver transplant (AALDLT) for vertically acquired genotype 1 hepatitis C-related cirrhosis. The early postoperative course was uneventful. She developed elevated liver chemistries and underwent a percutaneous liver biopsy demonstrating histologic recurrence of HCV within 8 months of transplant. She underwent 2 courses of therapy with pegylated interferon alfa-2b and ribavirin. She relapsed after the first course of 48 weeks of therapy; she was retreated shortly after that with the same regimen for 17 months and subsequently achieved a sustained virologic response. She did well with no changes in immune suppression or the development of other medical issues for 5 years post transplant (3 years after completion of HCV therapy). At that point, aminotransferases increased for unclear reasons (aspartate aminotransferase (AST) 106 U/L, alanine aminotransferase (ALT) 177 U/L) from a baseline of less than 30 U/L. Hepatitis C PCR tests, using a qualitative lower limit of detection of 50 IU/mL, were repeatedly negative. Due to the unexplained elevation in aminotransferases she was scheduled for liver biopsy. The only medications at that time were tacrolimus and Ortho Tri-Cyclen, and the patient denied herbal or alternative medications. Relevant additional past medical history was significant only for previous short segment small bowel resection for possible inflammatory bowel disease; this event was not temporally related to the elevated enzymes and histology revealed nonspecific bowel inflammation. 

Percutaneous ultrasound-guided right lobe liver biopsy was performed using a 15-guage core biopsy device by an experienced radiologist. Prior to the procedure, the patient was noted to be hypertensive with systolic blood pressure of 160–190 and diastolic pressures of 100–120. This was attributed to anxiety and her pressure normalized with an anxiolytic. The procedure was performed in the morning and was uneventful, and the patient's vital signs were normal after the biopsy. She was discharged home after a four-hour observation period. Histology revealed nonspecific mild portal inflammation, no fibrosis, and minimal endotheliitis and rare eosinophils raising the possibility of mild acute cellular rejection.

**Figure 1:**
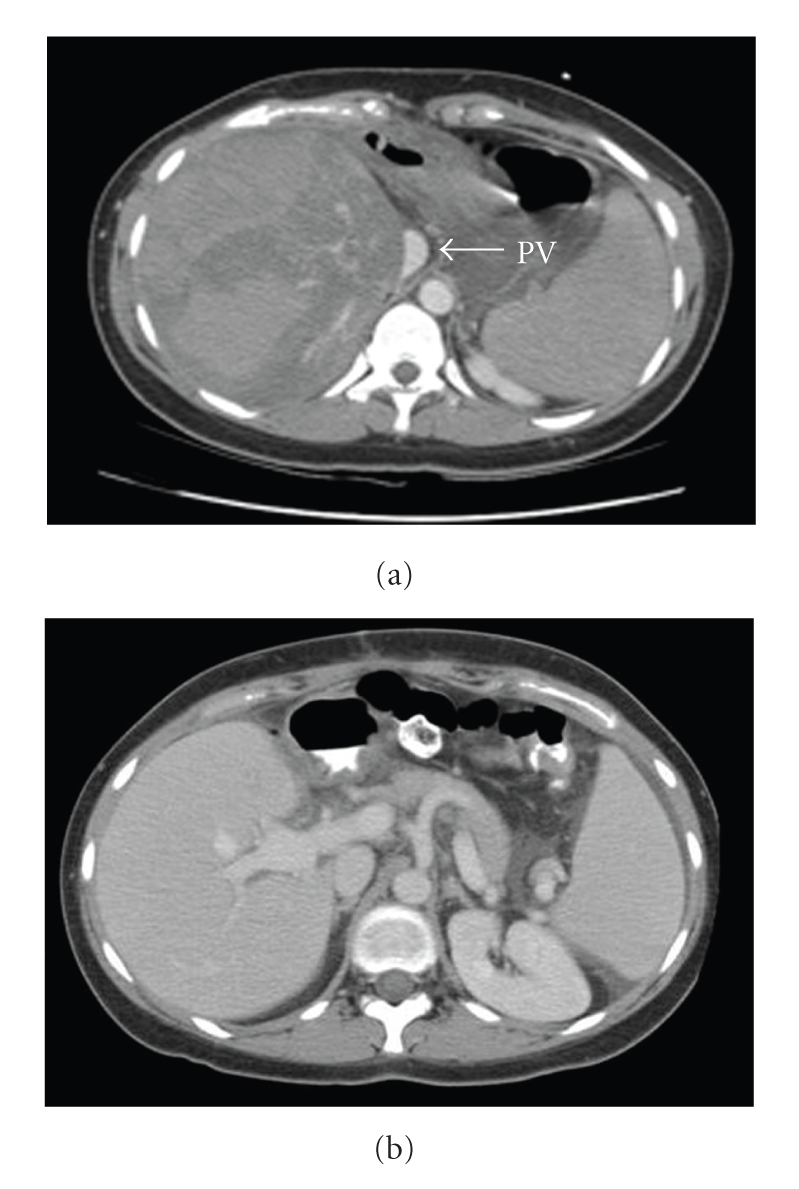
(a) CT scan demonstrating extensive intraparenchymal hematoma and compression of portal vein (PV) branches; (b) for comparison, a CT scan from the same patient from 2 years prior shows the normal appearance of a right lobe graft with normal portal vein branches.

The evening of the same day the patient developed mild right shoulder pain. The pain awakened her at 4 a.m. (roughly 20 hours post biopsy) and at this point was severe, in the shoulder and right upper quadrant, and accompanied by nausea and vomiting. On readmission through the emergency department, she had normal vital signs and her exam was notable for tenderness over the right upper quadrant. Laboratory values were notable for AST 810 U/L, ALT 772 U/L, alkaline phosphatase 227 U/L, bilirubin 1.8 mg/dL, prothrombin (PT) INR 1.0, hemoglobin 14.6 g/dL, hematocrit 41.3%, platelets 109,000/UL, BUN 18 mg/dL, and creatinine 0.7 mg/dL. No free air was noted on plain abdominal X-rays. A computerized tomography (CT) scan of the abdomen revealed a hyperdense mass within the right lobe of the liver, measuring 7.7 × 5.7 cm in diameter, traversing segments 6 and 7 and bordering on segment 8. In addition, there was a hypodense region within segment 8 in a subcapsular distribution which was felt to also represent hemorrhage or an area of infarction of the liver (Figure 1).

**Figure 2:**
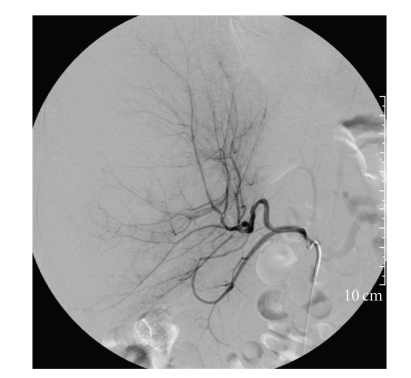
Hepatic arteriogram demonstrating medial displacement of hepatic arterioles due to mass effect of hematoma and lack of any arterial bleeding site.

Approximately 12 hours after admission (30 hours post biopsy), hemoglobin was 11.9 g/dL, AST 4947 U/L, ALT 4447 U/L, total bilirubin 4.3 mg/dL, and PT INR 2.1. She was transferred to the Medical Intensive Care Unit. Repeated CT scan demonstrated a slight increase in the anterolateral subcapsular hematoma, with decreased density of the parenchyma, and flattening and effacement of the inferior vena cava and portal veins consistent with compression by the enlarging hematoma. A celiac arteriogram was performed and revealed that the anterior and posterior branches of the right hepatic artery were attenuated and displaced medially due to large hematoma in the right lobe of the liver, but the hepatic artery was patent. Selective angiograms in the anterior and posterior branches of the right hepatic artery failed to show any active bleeding or pseudoaneurysm (Figure 2).

Within 24 hours of admission her clinical picture changed; she developed encephalopathy, tachypnea, and a decrease in hemoglobin to 8.9 g/dL. Other pertinent laboratory values included AST > 6000 U/L, ALT > 8000 U/L, total bilirubin 4.8 mg/dL, INR 2.2, Cr 1.7 mg/dL, and a pH 7.07. She was intubated and a bicarbonate drip was started to correct the acidosis. Over the next 24 hours, the PT INR rose to 5.5, total bilirubin increased to 15.5 mg/dL, and her mental status and acidosis worsened. She required 16 units of packed red blood cells, 24 units of fresh frozen plasma, hemodynamic support with pressors, and hemodialysis for acute renal failure. She was listed emergently for liver transplantation. 

**Figure 3:**
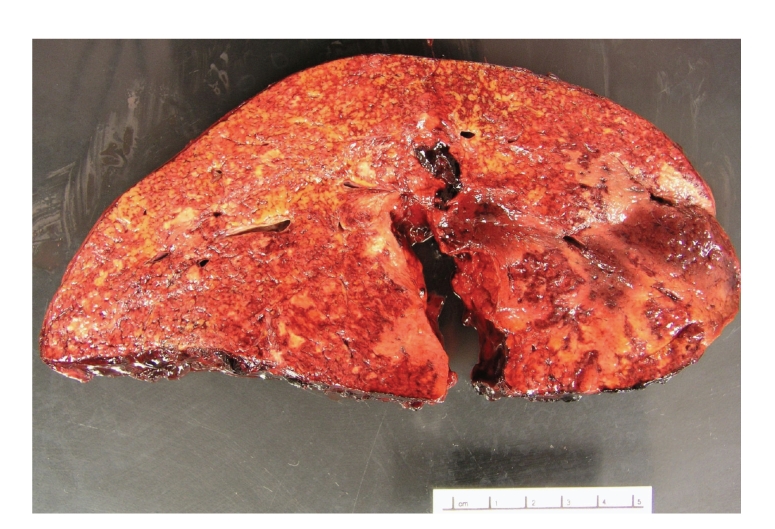
Gross photo of explanted liver demonstrating large area of hemorrhage and necrosis.

**Figure 4:**
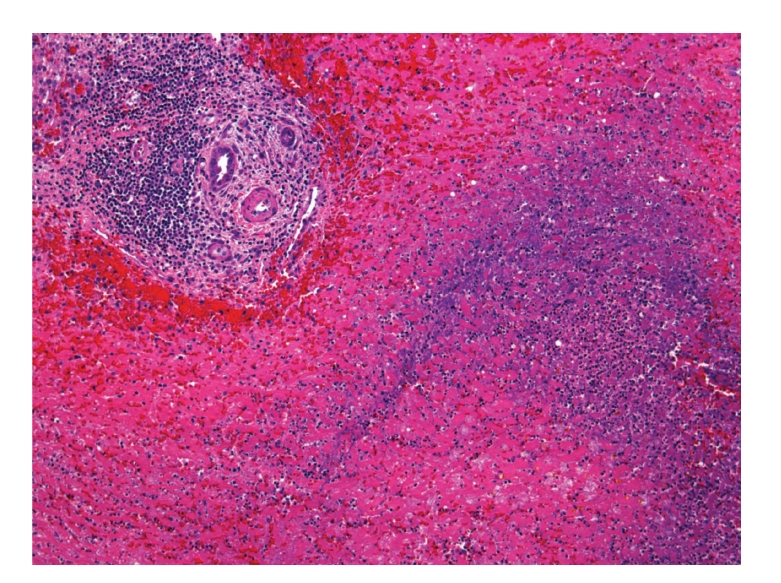
Photomicrograph of explanted liver demonstrating massive necrosis and architectural collapse.

Approximately 72 hours after admission she underwent orthotopic liver transplantation (OLT). Her liver was densely adherent to the abdominal wall and had a large hematoma along the capsule, extending over 15 cm of the surface, creating a firm and immobile liver. The main hepatic artery had evidence of acute thrombus (which was not present at angiogram). Explant demonstrated hemorrhage and adhesions of the serosa, multiple areas of ischemic necrosis occupying greater than 90% of the parenchyma, focal acute parenchymal hemorrhage, and central hematoma (Figures 3 and 4). After transplantation the patient's liver tests normalized, her renal failure improved, and she was discharged home on postoperative day 13.

## 3. Discussion

Percutaneous liver biopsy is considered a relatively safe procedure with an overall complication rate of 0.9%–3.7% with the most feared complication being that of major hemorrhage [1]. The risk of bleeding ranges from 0.06% to 1.7% and the risk of death (generally from bleeding) from 0.009% to 0.48% [4, 6–9]. Posttransplant biopsies have similar complication rates [10, 11].

Significant bleeding after liver biopsy is typically in the form of hemoperitoneum (0.32%) as opposed to intrahepatic hematoma (0.023%) [4, 5]. The incidence of mild asymptomatic bleeding is significantly higher than detected bleeding. Minuk et al. reported small hematomas seen via ultrasound in 23% of patients one day after biopsy [3]. Two prospective series have demonstrated intrahepatic hematomas on liver scan in 2% to 7% of subjects after biopsy [12, 13]. Vascular defects such as arteriovenous shunts and occluded arteries have also been reported. In 1976, Hellekant showed angiographic defects in 61% of patients within 1 week of biopsy, but in only 11% of angiograms done after one week [14]. Subsequent studies of angiography after percutaneous liver biopsy have shown arteriovenous fistulae, mostly arterioportal, in 5.4%–6.3% of patients within one week to one month of the procedure [15, 16]. Most arterioportal fistulae are asymptomatic and close spontaneously [16].

Complications of liver biopsy typically manifest within 24 hours, with 61% occurring within 2 hours and 96% becoming evident within 24 hours [4]. Bleeding generally results from laceration of Glisson's capsule or intrahepatic hematoma. Increased risk of bleeding is associated with older age, underlying malignancy, cirrhosis, and increasing number of biopsy passes [4, 6]. Hemoperitoneum is associated with a higher death rate in those with malignancy or cirrhosis [4, 6]. 

There are few published reports of intrahepatic hematomas resulting in death following liver biopsy [17]. Ahmed et al. report a case of hemorrhage from a hepatic artery pseudoaneurysm after liver biopsy. Bleeding continued despite embolization of the actively bleeding right hepatic arterial branch at initial angiography. Subsequent angiography showed new areas of active bleeding and laceration of the right hepatic lobe. Surgical oversewing and packing of the laceration were performed at laparotomy; however, the patient succumbed to multiorgan failure. Autopsy revealed foci of necrosis throughout both lobes of the liver, with the right lobe being almost entirely necrotic [18]. 

This is the first reported case of percutaneous liver biopsy resulting in a rapidly expanding hematoma with subsequent vascular compromise and acute liver failure. The initial CT scan within 24 hours of biopsy demonstrated portal vein compression. Hepatic arteriogram demonstrated patent hepatic arterial flow, but, 48 hours later, at the time of liver transplantation the hepatic artery had thrombosed. We postulate that the expanding hematoma in the presence of an intact hepatic capsule led to climbing intrahepatic pressure. This in turn caused pressure necrosis of hepatic parenchyma and compromised portal flow, while increased vascular resistance and vasoconstriction likely led to hepatic artery thrombosis. We believe this syndrome should be termed “Hepatic Compartment Syndrome” due to its similarity to the well-described abdominal compartment syndrome. In abdominal compartment syndrome, increased abdominal pressure leads to compromised perfusion through mechanisms including both increased pressure on abdominal viscera and vessels compromised by cardiac output. Similarly in hepatic compartment syndrome, it is likely that hepatic parenchymal pressure, hepatic necrosis, and hypovolemia work in concert to compromised hepatic perfusion. 

It is noteworthy that this condition developed in the setting of prior right lobe live donor liver transplant. Transplanted livers typically develop a dense adherence to the surrounding diaphragm and peritoneal surfaces which usually is only relevant if the patient requires retransplantation. In this case, the inability of the thickened capsule to expand likely contributed to the compartment syndrome. Transplanted livers may be more sensitive to blood flow compromise than the native liver either by the loss of vascular autoregulation or the loss of collateral flow. Additionally, a partial liver graft may be even more susceptible to vascular compromise, particularly hepatic artery thrombosis, due to the lack of a large contralateral vascular bed to allow for lateral shunting in cases of increased resistance. This is speculative; however, no data exists to support this. 

In summary, the constellation of rapidly expanding intrahepatic hematoma with hepatic vascular compromise and hepatic necrosis define hepatic compartment syndrome. The case presented resulted in fulminant hepatic failure requiring retransplantation, but certainly lesser degrees of hepatic injury could result. Whether this rare phenomenon is more likely to occur after either whole or live donor liver transplantation remains unknown. 

Percutaneous biopsy remains an extremely safe and important tool for managing the transplant patient, as severe bleeding or complication is rare. Keys to management include early recognition, hepatic angiogram to seek a bleeding arterial source, hemodynamic support and, as in this case, timely liver transplantation when hepatic failure is irreversible.
